# Predicting host tropism of influenza A virus proteins using random forest

**DOI:** 10.1186/1755-8794-7-S3-S1

**Published:** 2014-12-08

**Authors:** Christine LP Eng, Joo Chuan Tong, Tin Wee Tan

**Affiliations:** 1 Department of Biochemistry, Yong Loo Lin School of Medicine, National University of Singapore, Singapore, 117599, Singapore; 2 Institute of High Performance Computing, A*STAR, Singapore, 138632, Singapore

**Keywords:** Influenza, zoonotic, machine learning, random forest, prediction, host range restriction, host tropism, bioinformatics

## Abstract

**Background:**

Majority of influenza A viruses reside and circulate among animal populations, seldom infecting humans due to host range restriction. Yet when some avian strains do acquire the ability to overcome species barrier, they might become adapted to humans, replicating efficiently and causing diseases, leading to potential pandemic. With the huge influenza A virus reservoir in wild birds, it is a cause for concern when a new influenza strain emerges with the ability to cross host species barrier, as shown in light of the recent H7N9 outbreak in China. Several influenza proteins have been shown to be major determinants in host tropism. Further understanding and determining host tropism would be important in identifying zoonotic influenza virus strains capable of crossing species barrier and infecting humans.

**Results:**

In this study, computational models for 11 influenza proteins have been constructed using the machine learning algorithm random forest for prediction of host tropism. The prediction models were trained on influenza protein sequences isolated from both avian and human samples, which were transformed into amino acid physicochemical properties feature vectors. The results were highly accurate prediction models (ACC>96.57; AUC>0.980; MCC>0.916) capable of determining host tropism of individual influenza proteins. In addition, features from all 11 proteins were used to construct a combined model to predict host tropism of influenza virus strains. This would help assess a novel influenza strain's host range capability.

**Conclusions:**

From the prediction models constructed, all achieved high prediction performance, indicating clear distinctions in both avian and human proteins. When used together as a host tropism prediction system, zoonotic strains could potentially be identified based on different protein prediction results. Understanding and predicting host tropism of influenza proteins lay an important foundation for future work in constructing computation models capable of directly predicting interspecies transmission of influenza viruses. The models are available for prediction at http://fluleap.bic.nus.edu.sg.

## Background

Influenza is one of the most well-known infectious diseases attracting attention worldwide. Seasonal influenza epidemics are the cause of over three million severe cases of illness and about 300,000 to 500,000 deaths yearly [1].There have also been four influenza pandemics since the 20^th ^century, infecting millions of people and killing hundreds of thousands globally [2]. Because of this, influenza has been the subject of intensive research in the past century. While much knowledge regarding the virus has been discovered, we are still no closer to having the ability to predict the next pandemic, such as in the case of 2009 H1N1 pandemic. Current understanding of influenza zoonotic transmission potential of novel strains still remains poorly understood. This poses a significant threat to public health, not knowing when or where the next pandemic would strike.

A large number of influenza A viruses naturally reside in avian species where they constantly circulate and evolve. Most influenza A viruses are restricted to their host species, having limited capability to cross species barrier and infect a new host. It is not rare, however, for a virus strain to acquire the capability to make that zoonotic leap [3,4]. This is highlighted by confirmed cases of human infections by highly pathogenic H5N1 viruses, and more recently, the H7N9 outbreak in China [5]. Analysis of the recent influenza H7N9 outbreak in China found the virus strain to be a reassortant from multiple mixed avian sources, causing infections via direct contact with poultry [6,7]. Similar to H5N1 strains, this further affirms the potential of avian influenza strains capable of directly infecting human, causing severe illnesses.

Species barrier limits influenza strains from freely infecting different host organisms as they must overcome host range restriction to adapt to a new host. One crucial determinant of host tropism is hemagglutinin (HA) receptor specificity, in particular, preference of specific species of sialic acid on host cells. Human strains predominantly recognize α2,6-sialic acid linkages while avian strains preferentially binds receptors of α2,3-sialic acid linkages [8-10]. Studies in influenza receptor specificity have shown that specific amino acid substitutions can alter receptor binding site and binding specificity, which in turn, alters receptor preference [11-13]. Another major determinant involves viral polymerase complex, more specifically, the PB2 subunit which has long been implicated in playing a crucial role in determining host tropism. A single amino acid residue in PB2 at position 627 was found to be sufficient to determine host range of influenza viruses [14-16]. Glutamic acid is found at position 627 in most of the avian strains, whereas replacing the amino acid with lysine enables viral replication in humans [17-22]. Furthermore, genomic signatures of both avian and human influenza viruses have also been explored by position-specific entropy profiles created by comparing both types of viruses [23]. Mutations on specific positions may render an avian strain capable of infecting humans. All these information play a part in further contributing to the understanding of host tropism of influenza viruses.

Information from the underlying molecular mechanism of host tropism would be useful in the construction of computational prediction models. A novel prediction model was first constructed by Qiang and Kou to discriminate between avian and human influenza A viruses based on molecular patterns in protein sequences [24]. The model employed a method based on wavelet packet decomposition transforming protein sequences into energy feature vectors for training an artificial neural network (ANN) model. Another recent prediction model constructed by Wang *et al*. made use of the avian and human genomic signatures discovered previously to also classify avian and human strains [25]. Position-specific entropy profiles of avian and human protein sequences were encoded with amino acid physicochemical properties and then trained with support vector machine (SVM). Both prediction models classify avian or human influenza strains based on compilation of six inner proteins of influenza A viruses, including one matrix protein (M1), nucleoprotein (NP), one non-structural protein (NS1), and three RNA polymerases (PA, PB1 and PB2). These prediction models could be of use in predicting interspecies transmission of influenza A viruses.

In our study, we further extended the prediction models to include all 11 influenza proteins for the prediction of host tropism. The 11 proteins include HA, neuraminidase (NA), NP, both matrix proteins (M1 and M2), both non-structural proteins (NS1 and NS2), as well as the rest of the viral polymerase proteins (PA, PB1, PB1-F2, and PB2). Prediction model for each individual influenza A protein was constructed to predict host tropism of the protein. In addition, a combined prediction model was also constructed using all 11 proteins for each strain. Similar to previous studies, the final model could classify between avian and human influenza A viruses from protein sequences, providing clues into the host range a novel influenza A strain might be predisposed to. This could be crucial in providing an early insight of novel strains capable of crossing species barrier, leading towards the prediction of interspecies transmission of influenza A viruses.

## Methods

### Influenza protein sequence dataset

A total of 67,940 influenza A protein sequences isolated from avian and human hosts were obtained from Influenza Research Database http://www.fludb.org/ in February 2014 [26]. Incomplete and duplicate sequences were removed to minimize bias in the machine learning training process. Strains isolated from avian samples were classified as negative samples while human-isolated samples were classified positive. The protein datasets were further divided into separate training and testing datasets, by randomly allocating 20 percent of the sequences as testing datasets. Further details can be found in Table 1, which depicts the total number of samples used in the training and testing of each prediction model. An additional file lists the distribution of various influenza subtypes used in the training and testing dataset for each protein [see Additional file 1]. In summary, 20,923 positive human samples and 30,548 negative avian samples were used in the training of machine learning classification models as well as 5,262 positive human samples and 7,668 negative avian samples as testing datasets for external validation of the models.

**Table 1:** Total number of positive and negative samples for protein datasets and combined dataset.

Dataset	Training dataset			Testing dataset		
	Positive samples	Negative samples	Total samples	Positive samples	Negative samples	Total samples
HA	5449	5261	10710	1344	1357	2701
M1	547	908	1455	135	219	354
M2	644	1038	1682	178	268	446
NA	3945	4315	8260	963	1051	2014
NP	1148	2140	3288	282	537	819
NS1	1706	2940	4646	418	748	1166
NS2	475	1157	1632	133	246	379
PA	2135	4067	6202	573	997	1570
PB1	1995	3189	5184	504	797	1301
PB1-F2	722	2206	2928	167	588	755
PB2	2157	3327	5484	565	860	1425
Combined	3272	3923	7195	799	989	1788

The construction of a combined model utilized sequences of all proteins. Only strains with complete sequences from all 11 proteins were included in the training dataset. A total number of 3,272 positive human samples and 3,923 negative avian samples were used in the final training of a combined prediction model as well as 799 positive samples and 989 negative samples used as external testing dataset.

### Transforming protein sequence into feature vectors

Composition of amino acids and amino acid physicochemical properties were extracted from protein sequences as feature vectors for the training of machine learning algorithms. Composition of each of the 20 standard amino acids was first computed, yielding 20 feature vectors. This was performed by calculating the frequency of each amino acid along the length of the entire protein sequence. These feature vectors represent the composition of each individual amino acid in the protein sequence.

The next step of transformation was performed using a method developed by Dubchak *et. al*., in which three descriptors: composition (C), transition (T), and distribution (D), were calculated to globally describe amino acid properties [27,28]. The original four amino acid properties, hydrophobicity, normalized van der Waals volume, polarity, and polarizability were included, along with two other properties: charge and solvent accessibility. These amino acid properties divide amino acids into three groups based on amino acid indices by Tomii and Kanehisa [29]. The global descriptors, CTD, can be calculated using the following equations:

$$\text{C}=\left(\frac{n_{1}\times 100}{N}\right),\;\frac{n_{2}\times 100}{N}\left(,\;\frac{n_{3}\times 100}{N}\right)$$

$$\text{T}=\left(\frac{T_{G_{1}G_{2}}\times 100}{N-1}\right),\;\frac{T_{G_{1}G_{3}}\times 100}{N-1}\left(,\;\frac{T_{G_{2}G_{3}}\times 100}{N-1}\right)$$

$$\text{D}\;\;=\;\;\left({\text{D}}_{\text{1}},\;{\text{D}}_{\text{2}},\;{\text{D}}_{\text{3}}\right),$$

$${\text{D}}_{\text{i}}=\left(\frac{P_{i0}\times 100}{N}\right),\;\frac{P_{i25}\times 100}{N},\;\frac{P_{i50}\times 100}{N},\;\frac{P_{i75}\times 100}{N},\;\left(\frac{P_{i100}\times 100}{N}\right)$$

Composition describes the percentage frequency of amino acid property groups within the sequence, while transition calculates the percentage of transits between amino acids of differing property groups and distribution, on the other hand, represents the percentage at which the first, 25%, 50%, 75% and 100% of amino acids of a particular property group within the sequence [30-32]. The composition calculated in this step refers to the composition of each amino acid property group, instead of the 20 standard amino acids. Based on these, 21 global descriptors were calculated for each amino acid property. In full, 146 amino acid feature vectors represent protein sequences in the training of individual prediction models for the proteins.

### Training machine learning classifiers

The first step of machine learning classification involves selecting the best algorithm most suited to classifying the datasets. Experiments on various machine learning classifiers were performed on the WEKA platform and machine learning algorithms taken into consideration were random forest, *k*-nearest neighbor (kNN), Naïve Bayes, support vector machines (SVM), and artificial neural networks (ANN) [33]. Preliminary training revealed random forest to be best suited for the training of the dataset. Random forest is an ensemble learning method containing a combination of decision tree classifiers. Random trees in the forest are grown through training of a bootstrapped sample in the dataset, and then by splitting leaf nodes in the trees using only a randomly selected subset of the entire feature space [34]. Random forest was chosen as the machine learning classifier to train all the prediction models.

All training of prediction models were conducted using 10-fold cross-validation. In 10-fold cross-validation, the entire dataset is divided into 9 training subsets and 1 testing subset. The training process would iterate 10 rounds using the 9 training subsets while reserving the last subset for testing. In this way, every sample in the dataset would be tested exactly once, to prevent the problem of overfitting.

### Parameter optimization

Parameter optimization is an important step in the training of machine learning classifiers. To achieve the best performance, parameters for the classifiers must be fine-tuned so that the most appropriate parameters for the training dataset are chosen. For each model, parameter optimization was first carried out using grid search approach to select for the best parameters to train the final model. The random forest parameters tuned were number of trees and number of features used in the training. Grid search exhaustively applies every parameter in a manually specified subset to select for parameters achieving the best performance. However, this poses another problem of defining the maximum threshold for grid search to scour. This is because generally, as the number of tree grows, there would be more features for the model to consider from, and thus would be better for the classifier. Despite that, there is a threshold with which the increasing number of trees would bring no significant performance gain, but in fact only serves to increase computational burden [35]. In view of this, a maximum of 150 trees and 22 features were specified for the grid search approach. Parameters optimized for each prediction model is shown in Table 2, and prediction models were constructed with these optimized number of trees and features. The optimized parameters shown in Table 2 demonstrate that maximum number of trees and features are not necessary for best performance of the prediction models.

**Table 2:** Random forest optimized parameters.

Model	Number of trees	Number of features
HA	150	21
M1	110	13
M2	140	17
NA	150	16
NP	40	15
NS1	50	20
NS2	100	14
PA	60	18
PB1	40	10
PB1-F2	150	13
PB2	40	16
Combined	40	22

### Feature selection for combined model

In the combined prediction model comprising all 11 proteins, dimensionality reduction was applied to reduce the number of feature vectors for the training of machine learning classifiers. This was achieved by feature selection approach using variable importance method in random forest. As the method was not available on WEKA, this step was performed using the randomForest package developed by Liaw and Wiener in the statistical software R [36,37]. The variables were ranked using mean decrease in Gini gain, which measures the quality of each variable split in the tree [34,38]. The top 15 features for each protein were selected for inclusion as feature vectors into the dataset for the combined prediction model.

### Performance model evaluation

Performance of prediction models were evaluated from a number of measures including prediction accuracy, sensitivity, specificity, area under the curve (AUC), as well as Matthew's correlation coefficient (MCC). Prediction accuracy measures of the overall accuracy of the classifier by calculating the number of correctly classified avian and human samples over the total number of samples in the dataset. Sensitivity and specificity summarize the accuracies of positive and negative predictions respectively where sensitivity calculates the ratio of samples correctly predicted among all positive human samples in the dataset and specificity describes the ratio of samples correctly predicted among all negative avian samples in the dataset. AUC on the other hand, gives the probability of correctly identified true positive samples over random noise in the dataset [39]. Lastly, MCC measures the correlation between observed and predicted samples of the binary classification.

## Results

### Comparison of machine learning algorithms

There are many machine learning algorithms capable of classification problems, each with its own merits and limitations, and each suited to different kinds of dataset. To fully maximize prediction performance, a suite of machine learning classifiers was tested on the WEKA machine learning platform. Results in Table 3 show preliminary prediction performance of various machine learning classifiers trained on HA dataset, including random forest, Naïve Bayes, kNN, SVM, and ANN. All classifiers performed similarly well, suggesting clear demarcation between avian and human HA proteins. This further affirms the distinct receptor binding specificity of avian and human HA proteins. Nevertheless, random forest outcompeted all other classifiers, achieving 98.58% prediction accuracy (AUC = 0.996; MCC = 0.972), and hence was chosen as the classifier to train the remaining prediction models for individual proteins.

**Table 3:** Comparison of machine learning classifiers.

Classifier	Accuracy	Sensitivity	Specificity	AUC	MCC
**Random forest**	**98.58**	**0.978**	**0.994**	**0.996**	**0.972**
Naïve Bayes	96.42	0.942	0.988	0.970	0.930
kNN	98.24	0.982	0.983	0.983	0.965
SVM	97.38	0.953	0.996	0.974	0.948
ANN	98.40	0.977	0.991	0.993	0.968

### Performance evaluation of individual protein prediction models

After optimizing the parameters for each protein dataset, prediction models for 11 individual influenza proteins were then constructed with random forest. The performance results of 10-fold cross-validation training for each individual protein prediction model can be found in Table 4. All models were shown to achieve outstanding predictive performance, the lowest being NS2 model with 96.57% accuracy (AUC = 0.980; MCC = 0.916), while HA prediction model achieved the best predictive performance of 98.62% accuracy (AUC = 0.998; MCC = 0.972). The high performance of all cross-validation prediction models constructed diminishes the likelihood of overfitting which could decrease the models' ability to predict from novel protein sequences in the future.

**Table 4:** 10-fold cross-validation performance on optimized parameters for prediction models.

Model	Accuracy	Sensitivity	Specificity	AUC	MCC
HA	98.62	0.986	0.993	0.998	0.972
M1	97.66	0.977	0.987	0.985	0.950
M2	96.73	0.967	0.973	0.989	0.931
NA	98.35	0.984	0.991	0.996	0.967
NP	97.51	0.975	0.979	0.992	0.945
NS1	97.48	0.975	0.981	0.992	0.946
NS2	96.57	0.966	0.971	0.980	0.916
PA	98.21	0.982	0.992	0.995	0.960
PB1	97.26	0.973	0.990	0.992	0.942
PB1-F2	97.99	0.980	0.987	0.992	0.945
PB2	98.29	0.983	0.992	0.995	0.964
Combined	99.72	0.997	0.999	0.999	0.994

The constructed prediction models were further independently validated with separate testing datasets and likewise performed well, as seen from Table 5. The lowest accuracy was that of M2 model at 97.09% (AUC = 0.993; MCC = 0.939) while the highest was achieved by HA model with 98.78% accuracy (AUC = 0.997; MCC = 0.976). The results further demonstrated the high predictive accuracy of all individual protein models, which reaffirm the models' ability for future prediction of host tropism of influenza proteins.

**Table 5:** Performance evaluation with separate testing dataset.

Model	Accuracy	Sensitivity	Specificity	AUC	MCC
HA	98.78	0.988	0.992	0.997	0.976
M1	97.18	0.972	0.984	0.984	0.940
M2	97.09	0.971	0.971	0.993	0.939
NA	98.56	0.986	0.987	0.998	0.971
NP	97.56	0.976	0.965	0.991	0.946
NS1	97.86	0.979	0.976	0.994	0.953
NS2	97.63	0.976	1.000	0.976	0.948
PA	97.52	0.975	0.991	0.995	0.947
PB1	97.23	0.972	0.988	0.994	0.942
PB1-F2	98.54	0.985	0.988	0.994	0.957
PB2	97.89	0.979	0.991	0.996	0.956
Combined	99.83	0.998	1.000	0.998	0.997

### Selected features representing each protein dataset

In constructing the combined prediction model for prediction of influenza virus host, feature vectors from all 11 proteins were used. The consolidation of all amino acid physicochemical properties from each protein dataset would result in a complex high-dimensional feature space, possibly including redundant features. Thus, dimensionality reduction was achieved by feature selection approach, selecting the most relevant feature vectors for each protein. As such, a total of 165 feature vectors represent sequences of all 11 proteins in a virus strain.

In the transformation of protein sequences into feature vectors, each amino acid physicochemical property was represented by 11 global descriptors. Some amino acid property stood out in which several of its descriptors were selected in the top 15, signifying the importance of that property in determining host tropism for the particular protein. Table 6 lists the top amino acid properties for each protein dataset, with top properties having high mean decrease in Gini gain shown. Several proteins including HA, NA, NS1, PA, PB1 and PB2 seem to have dominant amino acid properties playing a major role in the classification of avian or human proteins.

**Table 6:** Top amino acid physicochemical properties identified using variable importance feature in random forest.

Model	Amino acid property	**AAIndex ID and reference **[29]
HA	Charge	KLEP940101 [53]
	Normalized van der Waals volume	FAUJ880103 [54]
	Polarizability	CHAM820101 [55]

NA	Solvent accessibility	JANJ780102/JAN780103 [56]
	Polarity	GRAR740102 [57]

NS1	Charge	KLEP940101 [53]

PA	Hydrophobicity	ENGD860101 [58]
	Polarity	GRAR740102 [57]

PB1	Solvent accessibility	JANJ780102/JAN780103 [56]

PB2	Charge	KLEP940101 [53]
	Solvent accessibility	JANJ780102/JAN780103 [56]

### Performance evaluation of combined proteins model

The combined prediction model was constructed from top features representing each protein sequence. In contrast with previous models predicting individual protein host tropism, the final prediction model was constructed to predict influenza virus host given an assortment of proteins of mixed origins.

Performance of cross-validation training of the combined model is also shown in Table 4. Surprisingly, prediction accuracy of the final model surpassed all individual protein models, achieving 99.72% accuracy (AUC = 0.999; MCC = 0.994). A separate independent testing dataset further validated the performance of the model by correctly classifying 99.83% of test instances (AUC = 0.998; MCC = 0.997), shown in Table 5. It is therefore evident that the combined model incorporating features of all proteins resulted in an improved prediction performance.

## Discussion

All 11 influenza protein prediction models demonstrated high predictive performance, capable of distinguishing between avian and human influenza proteins. This suggests that apart from HA and PB2, the remaining nine influenza proteins also show clear distinctions in avian and human host tropism. However, the roles these remaining nine proteins play in determining host tropism are yet unclear. What is further unknown is that how many proteins it would take to tip the scale rendering an avian strain acquiring the capability to cross species barrier and infect humans. Further research would be needed to determine the role they play in host tropism. But this first step in constructing individual protein prediction models would come in useful for future work in directly predicting interspecies transmission of influenza virus.

### Important amino acid physicochemical properties in host tropism

In the long evolutionary history of influenza, virus transfers between different host species allowed gene segments to be mixed, producing reassortant strains with both avian and human segments. This process might potentially enhance viral pathogenicity, allowing reassortant strains to adapt to new host species. Three of the four influenza pandemics that occurred since the 20^th ^century have been shown to be generated from reassortment among avian and human strains [2,25,40,41]. As different proteins may play a part in increasing or decreasing the species barrier for novel strains to cross, it would be beneficial to predict host tropism of each individual protein. This would aid in further understanding the complex interplay between various components in an influenza strain.

The feature selection process might have revealed important amino acid physicochemical properties determining host tropism of individual proteins. Interestingly, the properties charge, normalized Van der Waals volume and polarizability carry higher weightage compared to other properties in the classification of HA host tropism. The initial responsibility of overcoming host species barrier falls on HA which determines entry into host cells by binding to sialylated glycan receptors on cell surface [42]. As mentioned, mutations can alter receptor binding specificity which changes receptor preference. Studies looking into glycan receptor specificity have found that electrostatic charge has a role in influencing receptor binding dynamics between HA and receptors on host cells [43]. In general, HA is positively charged while glycan receptor on host cell is negatively charged [44]. Thus increasing or decreasing net charge of HA would alter electrostatic interactions which in turn affect binding affinity. This was demonstrated by studies which show that amino acid substitutions increasing or decreasing charge respectively enhance or reduce receptor binding affinity and avidity [45,46]. Another study looking into molecular dynamics between HA and human receptor have found that mutations in HA affects the binding free energy involving electrostatic and non-polar interactions [47]. Polarizability, which concerns a molecule's ability to be polarized, would therefore play a part in determining the binding interaction of HA and human receptor. Further, glycan topology has also been thought to critically influence receptor binding of avian and human strains. Interaction between HA and glycan receptor were found to be influenced by electrostatic charge and Van der Waals volume, causing glycans to adopt distinct topological profiles [48]. These changes no doubt affect the binding of HA to glycan receptors on cell surface, demonstrating the importance of these selected amino acid properties in determining the switch in species-specificity.

Yet another heavily investigated influenza protein is PB2, where the two top amino acid properties chosen, charge and solvent accessibility, corroborate with previous molecular and protein structure studies. The crystal structure of an independently folded domain elucidated from PB2 revealed that the critical residue 627 is positioned in the middle of a surface exposed to solvent [17,49]. The glutamic acid preferred by avian strains forms a negatively charged region which lysine disrupts and additionally establishes a region of positive charge on the surface [17,49]. Mutations in the region therefore appear to affect polymerase activity. Evidently, both charge and solvent accessibility features play an important part in classification of host tropism by the PB2 prediction model.

While the roles other proteins play in determining host tropism are less well characterized, and studies looking into molecular dynamics of these proteins are even fewer, differences in amino acid residues of avian and human strains can be interpreted to changes in these amino acid properties. For the other two viral polymerases, PA and PB1, differences between avian and human sequences would inadvertently affect polymerase activity determining host range. How these changes play a part in host range determination is still unknown, but the amino acid properties hydrophobicity, polarity and solvent accessibility seem to suggest involvement of interaction with host proteins. Another protein is NS1, which is known to function as a potent viral antagonist of host interferon response [17,50]. Studies have increasingly shown NS1 to be involved in host range determination, yet the mechanism remains unclear. However, it has been hypothesized that NS1 proteins from different strains have varying efficiency in interferon control, which could contribute to restriction in host range [50,51]. Functional or structural variations would therefore also translate to difference in the charge of the protein, distinguishing avian and human NS1 proteins. Therefore, even minute change in protein structure and function could tip the balance towards adaptation in avian or human host. Further molecular studies are therefore needed to investigate how these properties play a part in host tropism of influenza viruses.

### Computational prediction models of influenza host

The previous two computational prediction models by Qiang and Kou [24] and Wang *et. al*. [25] were successful in the classification of avian and human strains. One drawback however, is the utilization of only six inner proteins of influenza. This method disregards the importance of the two influenza glycoproteins, HA and NA which unquestionably play a huge role in determining host tropism. Removing them from consideration in the prediction process would not represent an accurate tropism of the entire strain. In contrast, the combined prediction model constructed in this study applies information from all 11 influenza proteins allowing a much more balanced representation of the virus strain. While the final prediction model is still short of directly predicting interspecies transmission of influenza viruses, it could provide an early insight into the host range a virus strain might be adapted to. The prediction models were implemented on a web server and are available for prediction online at http://fluleap.bic.nus.edu.sg.

### Protein prediction models as host tropism prediction system

Together, all 11 protein prediction models can be used as a host tropism prediction system. The prediction system is demonstrated below with eight selected sample strains, detailed in table 7. Four avian strains as well as four human strains of various influenza subtypes were selected. It should be noted that these strains were manually selected and meticulously checked against the training and testing datasets to ensure that they were not used in the construction of the prediction models nor the independent testing stage. Hence, the prediction results were not biased in any way as all eight strains were novel and not previously encountered by the prediction models.

**Table 7:** Further information on sample strains used in the demonstration of host tropism prediction system.

**No**.	Strain	Subtype	Country	Collection Year	Host
1.	A/turkey/England/50-92/1991	H5N1	United Kingdom	1991	Turkey
2.	A/wild duck/Korea/SH19-50/2010	H7N9	South Korea	2010	Duck
3.	A/Chicken/Hong Kong/220/97	H5N1	Hong Kong	1997	Chicken
4.	A/chicken/Shanghai/S1078/2013	H7N9	China	2013	Chicken
5.	A/Hong Kong/542/97	H5N1	Hong Kong	1997	Human
6.	A/Shanghai/01/2014	H7N9	China	2014	Human
7.	A/New York/231/2003	H1N2	USA	2003	Human
8.	A/Guangdong/ST798/2008	H3N2	China	2008	Human

**Figure 1:**
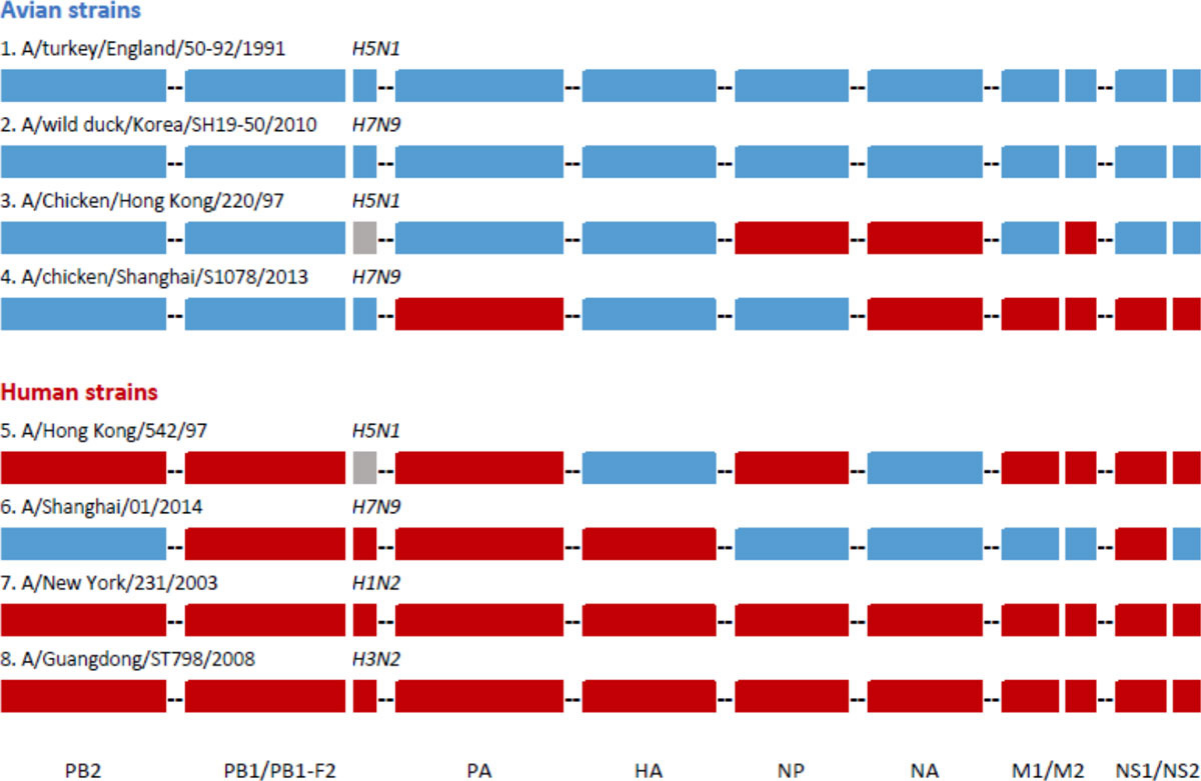
**Host tropism prediction results for sample strains**. The results for four avian strains are shown at the top while the bottom half shows results for four human strains. The prediction results were strung together illustrating an entire influenza A genome with eight segments encoding 11 proteins. The proteins coded by the segment are listed at the bottom of the figure. Each protein prediction is independent and is not influenced by prediction of other proteins. Blue bars represent a prediction of avian by the corresponding protein prediction model while red bars represent a prediction result of human. Grey bars indicate that prediction was not made as the corresponding protein sequence was not available or incomplete. Accurate predictions were made for all 11 proteins for the first two avian strains as well as the final two human strains. However, prediction results for the remaining four strains from the 1997 H5N1 outbreak in Hong Kong and the 2013 H7N9 outbreak in China show mixed predictions of avian and human proteins. The human strains isolated during the two outbreaks showing some of its proteins predicted as avian indicate the source of infection as most likely avian. On the other hand, the avian strains from chickens during the two outbreaks have several proteins that were predicted human and suggest that these proteins could have adapted to human host.

The host tropism prediction results for all eight strains are illustrated in Figure 1. Predictions for two human strains, *A/New York/231/2003 *and *A/Guangdong/ST798/2008 *were made accurately for all 11 proteins. These two strains are of influenza subtypes common to human, periodically circulating worldwide and infecting humans during annual flu season [4]. Hence, all of their proteins have adapted well in humans and were correctly predicted by the system. Likewise, accurate predictions for all 11 proteins were also made for two avian strains, *A/turkey/England/50-92/1991 *and *A/wild duck/Korea/SH19-50/2010*. The two avian strains of subtypes H5N1 and H7N9 were isolated from turkey and wild duck before the occurrence of these subtypes in humans. All 11 of their proteins were clearly avian proteins which again, were correctly predicted by the system. Prediction results for these two avian and human strains demonstrate the high accuracy of the host tropism prediction system, where despite making each protein prediction independently and not being influenced by other predictions, it is able to classify all proteins in each strain correctly.

However, the prediction results for the remaining four strains showed mixed avian and human prediction results. This could be attributed to error made in the prediction, or it could shed a whole new light on the capability of the host tropism prediction system. In 1997, the first human infection of influenza subtype H5N1 occurred in Hong Kong [52]. *A/Hong Kong/542/92 *was one of the strains isolated from a human patient during the outbreak. The prediction results showed most of its proteins predicted correctly as human, except HA and NA, which were predicted to be avian. This indicated that the virus strain most probably originated from an avian source as not all of its proteins have adapted to human. Prediction results of an avian strain, *A/Chicken/Hong Kong/220/97 *isolated from chicken during the same period also showed both avian and human proteins in the strain. The prediction results suggest that three of its proteins (NP, NA and M2) have either reassorted or mutated sufficiently to have adapted to humans, and more closely resemble human proteins.

In another case of novel human infection by an influenza subtype previously only found in birds, the prediction results for avian and human strains during the 2013 H7N9 outbreak in China also show similar mixed proteins host tropism signature. An avian strain isolated from chicken during the outbreak showed as many as six (PA, NA, M1, M2, NS1 and NS2) out of 11 proteins predicted to be human instead of the correct avian classification. It is more likely for the six proteins to resemble more closely to human proteins that the system was able to detect instead of prediction error, especially considering that the strain was isolated during the outbreak. A human strain from Shanghai, *A/Shanghai/01/2014 *isolated during the outbreak had five of its proteins predicted to be avian (PB1, PB1-F2, PA, HA, and NS1). This seem to suggest that only five human proteins, PB1, PB1-F2, PA, HA and NS1 were sufficient for the strain to have acquired the capability to escape its primary avian host and successfully infect humans. While it is still unclear which proteins are critical for an avian strain to acquire zoonotic capability and infect a new human host, this result could pave way for future work to perhaps answer the question as to how many proteins are required for a zoonotic avian strain to infect humans.

Ironically, the strength of this host tropism prediction system lies not in its almost perfect prediction accuracy, but rather when it makes a mistake in classifying avian or human proteins. The different types of prediction results shown above demonstrate the capability of the prediction system to detect potential zoonotic avian strains. Prediction results for strains isolated during the 1997 H5N1 Hong Kong outbreak and 2013 H7N9 China outbreak showed predictions of mixed avian and human proteins, having distinct host tropism protein signature that distinguishes them from typical avian or human strains. The prediction system is able to detect when individual proteins within a strain differ from its primary host. This showed tremendous potential of the prediction system in influenza surveillance whereby influenza strains can be continuously monitored to detect potentially zoonotic strains in avian that has yet to emerge in humans.

## Conclusion

The prediction models constructed for all influenza proteins show that besides HA and PB2 which are thought to be major determinants of host tropism, clear distinctions distinguish avian and human tropism for the remaining nine proteins. This study provides individual prediction models for all 11 influenza proteins determining host tropism and weighing the contribution of each protein in the eventual judgment of a novel virus strain's capability to cross species barrier. The prediction model combining all 11 proteins provides a first insight into a virus strain's host tropism, which might be useful as an early warning of its host range capability. When the prediction models are used together as a host tropism prediction system, zoonotic strains displayed mixed avian and human protein prediction results, distinct from typical avian or human strains. Based on protein prediction results alone, the host tropism prediction system might be able to identify zoonotic strains. Only by first understanding the underlying host tropism can promiscuous virus strains having the capability to cross species barrier be identified. With this foundation of host tropism prediction models, future work can be focused on building stronger computational models predicting direct avian-to-human transmission of influenza viruses. This would be a valuable tool in future surveillance of potentially hazardous influenza virus strains.

## List of abbreviations used

ANN: Artificial neural network; AUC: Area under the curve; C: Composition; D: Distribution; HA: Hemagglutinin; kNN: k-nearest neighbor; M1: Matrix protein 1; M2: Matrix protein 2; MCC: Matthew's correlation coefficient; NA: Neuraminidase; NP: Nucleoprotein; NS1: Non-structural protein 1; NS2: Non-structural protein 2; PA: RNA polymerase PA subunit; PB1: RNA polymerase PB1 subunit; PB1-F2: PB1-F2 protein; PB2: RNA polymerase PB2 subunit; SVM: Support vector machine; T: Transition

## Competing interests

TWT acted as an editor of the supplement in which this article appears, but in accordance with the journal's peer review requirements, did not act as an editor with respect to this article. The remaining authors declare that they have no competing interests.

## Authors' contributions

CLPE designed the project, constructed the prediction models and drafted the manuscript. JCT and TWT provided supervision and critical insights to the project as well as significant input to the manuscript. All authors read and approved the final manuscript.

## Supplementary Material

Additional file 1 **Supplementary materials**. The file lists subtype distribution of samples in each protein dataset. The influenza A subtype distribution for each protein is listed in a table, separated into training and testing datasets, and ordered from largest to smallest. Click here for file

## Declarations

Funding for publication of this article was provided by the Department of Biochemistry, National University of Singapore.

This article has been published as part of *BMC Medical Genomics *Volume 7 Supplement 3, 2014: Thirteenth International Conference on Bioinformatics (InCoB2014): Medical Genomics. The full contents of the supplement are available online at http://www.biomedcentral.com/bmcmedgenomics/supplements/7/S3.

## References

[B1] Influenza (seasonal) fact sheet no. 211 http://www.who.int/mediacentre/factsheets/fs211/en/

[B2] Kilbourne ED Influenza pandemics of the 20th century Emerg Infect Dis 2006 12 1 9 14 10.3201/eid1201.051254 16494710 PMC3291411

[B3] Kuiken T Holmes EC McCauley J Rimmelzwaan GF Williams CS Grenfell BT Host species barriers to influenza virus infections Science 2006 312 5772 394 397 10.1126/science.1122818 16627737

[B4] Medina RA Garcia-Sastre A Influenza A viruses: new research developments Nat Rev Microbiol 2011 9 8 590 603 10.1038/nrmicro2613 21747392 PMC10433403

[B5] Avian influenza A(H7N9) virus http://www.who.int/influenza/human_animal_interface/influenza_h7n9/en/

[B6] Liu D Shi W Shi Y Wang D Xiao H Li W Bi Y Wu Y Li X Yan J Origin and diversity of novel avian influenza A H7N9 viruses causing human infection: phylogenetic, structural, and coalescent analyses Lancet 2013 381 9881 1926 1932 10.1016/S0140-6736(13)60938-1 23643111

[B7] Wang Y Dai Z Cheng H Liu Z Pan Z Deng W Gao T Li X Yao Y Ren J Towards a better understanding of the novel avian-origin H7N9 influenza A virus in China Sci Rep 2013 3 2318 23897131 10.1038/srep02318 PMC3727058

[B8] Matrosovich MN Gambaryan AS Teneberg S Piskarev VE Yamnikova SS Lvov DK Robertson JS Karlsson KA Avian influenza A viruses differ from human viruses by recognition of sialyloligosaccharides and gangliosides and by a higher conservation of the HA receptor-binding site Virology 1997 233 1 224 234 10.1006/viro.1997.8580 9201232

[B9] Rogers GN Paulson JC Receptor determinants of human and animal influenza virus isolates: differences in receptor specificity of the H3 hemagglutinin based on species of origin Virology 1983 127 2 361 373 10.1016/0042-6822(83)90150-2 6868370

[B10] Suzuki Y Gangliosides as influenza virus receptors. Variation of influenza viruses and their recognition of the receptor sialo-sugar chains Prog Lipid Res 1994 33 4 429 457 10.1016/0163-7827(94)90026-4 7870741

[B11] Daniels RS Douglas AR Skehel JJ Wiley DC Naeve CW Webster RG Rogers GN Paulson JC Antigenic analyses of influenza virus haemagglutinins with different receptor-binding specificities Virology 1984 138 1 174 177 10.1016/0042-6822(84)90158-2 6208680

[B12] Yamada S Suzuki Y Suzuki T Le MQ Nidom CA Sakai-Tagawa Y Muramoto Y Ito M Kiso M Horimoto T Haemagglutinin mutations responsible for the binding of H5N1 influenza A viruses to human-type receptors Nature 2006 444 7117 378 382 10.1038/nature05264 17108965

[B13] Newhouse EI Xu D Markwick PR Amaro RE Pao HC Wu KJ Alam M McCammon JA Li WW Mechanism of glycan receptor recognition and specificity switch for avian, swine, and human adapted influenza virus hemagglutinins: a molecular dynamics perspective J Am Chem Soc 2009 131 47 17430 17442 10.1021/ja904052q 19891427 PMC2782351

[B14] Li OT Chan MC Leung CS Chan RW Guan Y Nicholls JM Poon LL Full factorial analysis of mammalian and avian influenza polymerase subunits suggests a role of an efficient polymerase for virus adaptation PloS one 2009 4 5 e5658 10.1371/journal.pone.0005658 19462010 PMC2680953

[B15] Jagger BW Memoli MJ Sheng ZM Qi L Hrabal RJ Allen GL Dugan VG Wang R Digard P Kash JC The PB2-E627K mutation attenuates viruses containing the 2009 H1N1 influenza pandemic polymerase mBio 2010 1 1 20689744 10.1128/mBio.00067-10 PMC2912665

[B16] Subbarao EK London W Murphy BR A single amino acid in the PB2 gene of influenza A virus is a determinant of host range J Virol 1993 67 4 1761 1764 8445709 10.1128/jvi.67.4.1761-1764.1993 PMC240216

[B17] Cauldwell AV Long JS Moncorge O Barclay WS Viral determinants of influenza A host range J Gen Virol 2014 95 1193 1210 10.1099/vir.0.062836-0 24584475

[B18] Chen H Bright RA Subbarao K Smith C Cox NJ Katz JM Matsuoka Y Polygenic virulence factors involved in pathogenesis of 1997 Hong Kong H5N1 influenza viruses in mice Virus Res 2007 128 1-2 159 163 10.1016/j.virusres.2007.04.017 17521765

[B19] Gao R Cao B Hu Y Feng Z Wang D Hu W Chen J Jie Z Qiu H Xu K Human infection with a novel avian-origin influenza A (H7N9) virus N Engl J Med 2013 368 20 1888 1897 10.1056/NEJMoa1304459 23577628

[B20] Hatta M Gao P Halfmann P Kawaoka Y Molecular basis for high virulence of Hong Kong H5N1 influenza A viruses Science 2001 293 5536 1840 1842 10.1126/science.1062882 11546875

[B21] Shinya K Hamm S Hatta M Ito H Ito T Kawaoka Y PB2 amino acid at position 627 affects replicative efficiency, but not cell tropism, of Hong Kong H5N1 influenza A viruses in mice Virology 2004 320 2 258 266 10.1016/j.virol.2003.11.030 15016548

[B22] Steel J Lowen AC Mubareka S Palese P Transmission of influenza virus in a mammalian host is increased by PB2 amino acids 627K or 627E/701N PLoS Pathog 2009 5 1 e1000252 10.1371/journal.ppat.1000252 19119420 PMC2603332

[B23] Chen GW Chang SC Mok CK Lo YL Kung YN Huang JH Shih YH Wang JY Chiang C Chen CJ Genomic signatures of human versus avian influenza A viruses Emerg Infect Dis 2006 12 9 1353 1360 17073083 10.3201/eid1209.060276 PMC3294750

[B24] Qiang X Kou Z Prediction of interspecies transmission for avian influenza A virus based on a back-propagation neural network Math Comput Model 2010 52 11-12 2060 2065 10.1016/j.mcm.2010.06.008

[B25] Wang J Ma C Kou Z Zhou Y Liu H Predicting transmission of avian influenza A viruses from avian to human by using informative physicochemical properties Int J Data Min Bioinform 2013 7 2 166 179 10.1504/IJDMB.2013.053198 23777174

[B26] Squires RB Noronha J Hunt V Garcia-Sastre A Macken C Baumgarth N Suarez D Pickett BE Zhang Y Larsen CN Influenza research database: an integrated bioinformatics resource for influenza research and surveillance Influenza Other Respir Viruses 2012 6 6 404 416 10.1111/j.1750-2659.2011.00331.x 22260278 PMC3345175

[B27] Dubchak I Muchnik I Holbrook SR Kim SH Prediction of protein folding class using global description of amino acid sequence Prod Natl Acad Sci USA 1995 92 19 8700 8704 10.1073/pnas.92.19.8700 7568000 PMC41034

[B28] Dubchak I Muchnik I Mayor C Dralyuk I Kim SH Recognition of a protein fold in the context of the Structural Classification of Proteins (SCOP) classification Proteins 1999 35 4 401 407 10.1002/(SICI)1097-0134(19990601)35:4<401::AID-PROT3>3.0.CO;2-K 10382667

[B29] Tomii K Kanehisa M Analysis of amino acid indices and mutation matrices for sequence comparison and structure prediction of proteins Protein Eng 1996 9 1 27 36 10.1093/protein/9.1.27 9053899

[B30] Cui J Han LY Lin HH Zhang HL Tang ZQ Zheng CJ Cao ZW Chen YZ Prediction of MHC-binding peptides of flexible lengths from sequence-derived structural and physicochemical properties Mol Immunol 2007 44 5 866 877 10.1016/j.molimm.2006.04.001 16806474

[B31] Li ZR Lin HH Han LY Jiang L Chen X Chen YZ PROFEAT: a web server for computing structural and physicochemical features of proteins and peptides from amino acid sequence Nucleic Acids Res 2006 34 Web Server W32 37 16845018 10.1093/nar/gkl305 PMC1538821

[B32] El-Manzalawy Y Dobbs D Honavar V On evaluating MHC-II binding peptide prediction methods PloS one 2008 3 9 e3268 10.1371/journal.pone.0003268 18813344 PMC2533399

[B33] Hall M Frank E Holmes G Pfahringer B Reutemann P Witten IH The WEKA data mining software: an update SIGKDD Explorations 2009 11 1 10 18 10.1145/1656274.1656278

[B34] Breiman L Random forests Mach Learn 2001 45 1 5 32 10.1023/A:1010933404324

[B35] Oshiro T Perez P Baranauskas J Perner P How many trees in a random forest? Machine Learning and Data Mining in Pattern Recognition 2012 7376 Springer Berlin Heidelberg 154 168 10.1007/978-3-642-31537-4_13

[B36] Liaw A Wiener M Classification and regression by randomForest R News 2002 2 3 18 22

[B37] R: A language and environment for statistical computing http://www.R-project.org/

[B38] Yang ZR World Scientific (Firm) Wang JTL Machine learning approaches to bioinformatics Science, Engineering, and Biology Informatics 2010 4 Singapore ; World Scientific Pub. Co 322

[B39] Hanley JA McNeil BJ The meaning and use of the area under a receiver operating characteristic (ROC) curve Radiology 1982 143 1 29 36 10.1148/radiology.143.1.7063747 7063747

[B40] Fang R Min Jou W Huylebroeck D Devos R Fiers W Complete structure of A/duck/Ukraine/63 influenza hemagglutinin gene: animal virus as progenitor of human H3 Hong Kong 1968 influenza hemagglutinin Cell 1981 25 2 315 323 10.1016/0092-8674(81)90049-0 6169439

[B41] Schafer JR Kawaoka Y Bean WJ Suss J Senne D Webster RG Origin of the pandemic 1957 H2 influenza A virus and the persistence of its possible progenitors in the avian reservoir Virology 1993 194 2 781 788 10.1006/viro.1993.1319 7684877

[B42] Skehel JJ Wiley DC Receptor binding and membrane fusion in virus entry: the influenza hemagglutinin Annu Rev Biochem 2000 69 531 569 10.1146/annurev.biochem.69.1.531 10966468

[B43] Arinaminpathy N Grenfell B Dynamics of glycoprotein charge in the evolutionary history of human influenza PloS one 2010 5 12 e15674 10.1371/journal.pone.0015674 21209885 PMC3012697

[B44] Gambaryan AS Matrosovich MN Bender CA Kilbourne ED Differences in the biological phenotype of low-yielding (L) and high-yielding (H) variants of swine influenza virus A/NJ/11/76 are associated with their different receptor-binding activity Virology 1998 247 2 223 231 10.1006/viro.1998.9274 9705915

[B45] Hensley SE Das SR Bailey AL Schmidt LM Hickman HD Jayaraman A Viswanathan K Raman R Sasisekharan R Bennink JR Hemagglutinin receptor binding avidity drives influenza A virus antigenic drift Science 2009 326 5953 734 736 10.1126/science.1178258 19900932 PMC2784927

[B46] Kobayashi Y Suzuki Y Compensatory evolution of net-charge in influenza A virus hemagglutinin PloS one 2012 7 7 e40422 10.1371/journal.pone.0040422 22808159 PMC3395715

[B47] Lee AN Hartono YD Sun T Leow ML Liu XW Huang X Zhang D Molecular dynamics studies of human receptor molecule in hemagglutinin of 1918 and 2009 H1N1 influenza viruses J Mol Modeling 2011 17 7 1635 1641 10.1007/s00894-010-0867-5 20978916

[B48] Xu D Newhouse EI Amaro RE Pao HC Cheng LS Markwick PR McCammon JA Li WW Arzberger PW Distinct glycan topology for avian and human sialopentasaccharide receptor analogues upon binding different hemagglutinins: a molecular dynamics perspective J Mol Biol 2009 387 2 465 491 10.1016/j.jmb.2009.01.040 19356594 PMC2892341

[B49] Tarendeau F Boudet J Guilligay D Mas PJ Bougault CM Boulo S Baudin F Ruigrok RW Daigle N Ellenberg J Structure and nuclear import function of the C-terminal domain of influenza virus polymerase PB2 subunit Nat Struct Mol Biol 2007 14 3 229 233 10.1038/nsmb1212 17310249

[B50] Carrillo B Choi JM Bornholdt ZA Sankaran B Rice AP Prasad BV The influenza A virus protein NS1 displays structural polymorphism J Virol 2014 88 8 4113 4122 10.1128/JVI.03692-13 24478439 PMC3993732

[B51] Hayman A Comely S Lackenby A Hartgroves LC Goodbourn S McCauley JW Barclay WS NS1 proteins of avian influenza A viruses can act as antagonists of the human alpha/beta interferon response J Virol 2007 81 5 2318 2327 10.1128/JVI.01856-06 17182679 PMC1865923

[B52] Centers for Disease Control and Prevention Isolation of avian influenza A(H5N1) viruses from humans--Hong Kong, May-December 1997 Morb Mortal Wkly Rep 1997 46 50 1204 1207 9414153

[B53] Klein P Kanehisa M DeLisi C Prediction of protein function from sequence properties. Discriminant analysis of a data base Biochim Biophys Acta 1984 787 3 221 226 10.1016/0167-4838(84)90312-1 6547351

[B54] Fauchere JL Charton M Kier LB Verloop A Pliska V Amino acid side chain parameters for correlation studies in biology and pharmacology Int J Pept Protein Res 32 4 269 278 3209351 10.1111/j.1399-3011.1988.tb01261.x

[B55] Charton M Charton BI The structural dependence of amino acid hydrophobicity parameters J Theor Biol 1982 99 4 629 644 10.1016/0022-5193(82)90191-6 7183857

[B56] Janin J Wodak S Conformation of amino acid side-chains in proteins J Mol Biol 1978 125 3 357 386 10.1016/0022-2836(78)90408-4 731698

[B57] Grantham R Amino acid difference formula to help explain protein evolution Science 1974 185 4154 862 864 10.1126/science.185.4154.862 4843792

[B58] Engelman DM Steitz TA Goldman A Identifying nonpolar transbilayer helices in amino acid sequences of membrane proteins Annu Rev Biophys Biomol Struct 1986 15 321 353 10.1146/annurev.biophys.15.1.321 3521657

