# Supplementary material for: Predicting host tropism of influenza A virus proteins using random forest
Source: BMC Med Genomics. 2014 Dec 8;7(Suppl 3):S1. doi: 10.1186/1755-8794-7-S3-S1 (PMC4290784; doi:10.1186/1755-8794-7-S3-S1)
Supplement: Additional file 1 — Supplementary materials. The file lists subtype distribution of samples in each protein dataset. The influenza A subtype distribution for each protein is listed in a table, separated into training and testing datasets, and ordered from largest to smallest. [file 1755-8794-7-S3-S1-S1.pdf]

Supplementary materials

Subtype distribution of samples in training and testing dataset of each protein

*Others represent subtypes which are incomplete, mixed or unknown.

HA

Training dataset		Testing dataset	
H1N1	3182	H1N1	809
H3N2	2212	H3N2	545
H5N1	1155	H5N1	292
H9N2	757	H9N2	174
H5N2	296	H5N2	82
H3N8	281	H4N6	72
H6N2	236	H3N8	67
Others*	214	H6N2	65
H4N6	210	Others*	58
H6N1	168	H7N3	40
H7N3	145	H10N7	37
H11N9	134	H6N1	36
H6N6	125	H11N9	35
H10N7	115	H6N6	28
H2N2	107	H2N3	27
H2N3	95	H7N2	24
H7N7	93	H6N8	23
H7N2	82	H7N7	19
H7N1	63	H4N8	17
H8N4	58	H8N4	16
H6N8	57	H5N3	15
H12N5	57	H2N2	14
H1N2	54	H7N1	14
H3N6	54	H1N2	11
H11N2	50	H3N6	10
H5N3	45	H12N5	10
H7N9	41	H11N2	9
H4N8	40	H1N3	7
H4N2	32	H7N9	7
H6N5	29	H2N9	6
H13N6	25	H6N9	6
H16N3	24	H13N6	6
H2N9	21	H3N3	5
H2N1	20	H4N2	5
H7N6	20	H5N9	5
H10N3	19	H6N5	5
H5N9	16	H10N1	5
H11N1	15	H11N1	5
H11N3	15	H2N7	4
H3N1	14	H3N1	4
H2N7	12	H10N2	4
H2N8	12	H1N5	3
H1N3	11	H1N9	3
H9N1	11	H2N1	3
H10N4	11	H2N8	3
H2N5	10	H7N6	3
H3N5	10	H9N6	3
H4N3	10	H13N2	3
H10N6	10	H13N9	3
H10N9	10	H2N5	2
H1N9	9	H4N3	2
H5N5	9	H4N9	2
H9N5	8	H5N7	2
H9N9	8	H8N2	2
H11N6	8	H9N1	2
H5N8	7	H9N7	2
H7N4	7	H10N4	2
H7N8	7	H10N8	2
H10N2	7	H11N3	2
H10N8	7	H11N6	2
H11N8	7	H12N8	2
H13N2	7	H16N3	2
H3N7	6	H1N8	1
H4N9	6	H3N5	1
H6N4	6	H3N7	1
H10N1	6	H3N9	1
H10N5	6	H4N1	1
H13N9	6	H4N5	1
H1N5	5	H4N7	1
H3N3	5	H5N4	1
H4N4	5	H5N5	1
H5N7	5	H5N6	1
H12N4	5	H6N3	1
H1N6	4	H7N4	1

H3N9	4	H7N5	1
H4N7	4	H7N8	1
H6N3	4	H8N5	1
H1N4	3	H9N3	1
H4N5	3	H9N5	1
H6N9	3	H9N9	1
H9N8	3	H10N3	1
H11N7	3	H10N5	1
H12N1	3	H10N6	1
H12N6	3	H11N7	1
H1N8	2	H11N8	1
H2N6	2	H12N3	1
H4N1	2	H12N9	1
H5N4	2	H14N6	1
H6N7	2	H15N4	1
H7N5	2	H15N8	1
H9N7	2		
H12N2	2		
H12N3	2		
H12N7	2		
H12N8	2		
H13N8	2		
H14N5	2		
H3N4	1		
H5N6	1		
H8N2	1		
H8N8	1		
H9N4	1		
H9N6	1		
H11N4	1		
H11N5	1		
H14N2	1		
H15N2	1		
H15N8	1		
H15N9	1		

M1

Training dataset		Testing dataset	
H1N1	297	H1N1	75
H3N2	236	H5N1	55
H5N1	220	H3N2	50
H9N2	182	H9N2	49
H5N2	55	H5N2	17
H6N2	53	H6N2	10
H6N1	39	H7N2	10
H7N3	29	H7N3	10
H7N2	28	H4N6	8
H3N8	27	H2N2	7
Others*	27	H6N1	7
H4N6	23	H11N9	7
H2N2	22	H7N1	6
H7N7	22	H6N6	4
H7N1	21	H7N9	4
H6N6	19	H3N8	3
H11N9	15	H4N8	3
H1N2	13	H6N8	3
H6N8	12	H10N7	3
H10N7	11	Others*	3
H6N5	9	H2N1	2
H4N8	8	H5N9	2
H13N6	8	H11N8	2
H3N6	7	H16N3	2
H5N3	6	H1N2	1
H4N2	5	H2N3	1
H7N9	5	H4N9	1
H10N4	5	H5N3	1
H2N3	4	H6N3	1
H5N5	4	H7N7	1
H11N2	4	H9N1	1
H11N3	4	H10N2	1
H8N4	3	H10N3	1
H11N1	3	H10N8	1
H2N7	2	H12N1	1
H2N9	2	H12N9	1
H6N9	2		
H10N8	2		
H11N6	2		
H12N5	2		
H1N3	1		
H1N4	1		
H2N6	1		

H3N1	1
H3N7	1
H5N7	1
H5N8	1
H5N9	1
H6N3	1
H6N4	1
H7N6	1
H10N1	1
H10N3	1
H11N7	1
H12N4	1
H13N9	1
H16N3	1

M2

Training dataset		Testing dataset	
H3N2	326	H1N1	85
H1N1	304	H3N2	85
H5N1	267	H5N1	66
H9N2	180	H9N2	48
H5N2	68	H5N2	20
H7N2	45	Others*	16
H6N1	43	H3N8	12
H6N2	42	H6N1	12
H3N8	37	H7N2	10
H4N6	32	H6N2	9
H7N3	32	H2N2	7
Others*	23	H4N6	7
H11N9	21	H6N6	5
H2N2	20	H4N8	5
H1N2	16	H3N6	4
H7N7	16	H6N8	4
H10N7	13	H7N9	4
H5N3	12	H1N2	3
H6N6	11	H7N1	3
H2N3	9	H7N3	3
H4N2	9	H7N7	3
H13N6	9	H10N7	3
H2N1	8	H12N5	3
H3N6	8	H2N3	2
H11N1	8	H2N7	2
H4N8	7	H5N5	2
H6N8	7	H7N6	2
H7N9	7	H8N4	2
H12N5	7	H11N2	2
H16N3	7	H13N9	2
H7N1	6	H2N8	1
H6N5	5	H2N9	1
H10N3	5	H3N3	1
H2N7	4	H4N2	1
H8N4	4	H4N3	1
H11N2	4	H4N9	1
H2N9	3	H5N3	1
H5N5	3	H5N8	1
H5N8	3	H6N5	1
H5N9	3	H10N8	1
H7N6	3	H11N3	1
H9N1	3	H11N9	1
H1N3	2	H13N2	1
H2N5	2	H13N6	1
H3N3	2	H13N8	1
H3N5	2		
H4N5	2		
H11N6	2		
H13N9	2		
H1N6	1		
H1N8	1		
H2N4	1		
H2N8	1		
H3N1	1		
H4N3	1		
H4N7	1		
H4N9	1		
H5N7	1		
H6N3	1		
H6N4	1		
H6N9	1		
H7N8	1		
H8N8	1		
H9N5	1		
H9N6	1		
H9N7	1		
H10N2	1		
H10N4	1		
H10N5	1		
H10N8	1		
H10N9	1		
H11N3	1		
H12N1	1		
H12N3	1		
H12N6	1		
H13N2	1		
H14N6	1		

NA

Training dataset		Testing dataset	
H1N1	2057	H1N1	482
H3N2	1837	H3N2	469
H5N1	900	H5N1	215
H9N2	489	H9N2	132
H5N2	251	H3N8	63
Others*	248	H6N2	58
H3N8	247	H4N6	53
H6N2	225	Others*	51
H4N6	220	H5N2	41
H6N1	164	H6N6	34
H11N9	107	H6N1	32
H6N6	98	H10N7	32
H7N3	98	H2N2	26
H10N7	98	H2N3	25
H2N2	91	H7N3	22
H7N2	85	H11N9	22
H2N3	78	H6N8	17
H7N7	63	H7N7	15
H6N8	58	H11N2	15
H3N6	55	H1N2	13
H12N5	46	H4N2	13
H4N8	44	H5N3	13
H11N2	44	H7N2	13
H1N2	38	H7N1	9
H7N1	38	H8N4	9
H7N9	38	H12N5	9
H8N4	32	H3N1	8
H5N3	31	H3N6	8
H10N3	27	H4N8	8
H6N5	25	H1N3	6
H2N9	23	H7N9	6
H3N1	21	H11N1	6
H2N1	19	H4N3	5
H4N2	16	H10N9	5
H7N6	16	H11N3	5
H1N3	15	H3N3	4
H16N3	15	H6N5	4
H13N6	13	H10N3	4
H11N3	12	H13N9	4
H1N9	11	H2N9	3
H5N5	9	H3N5	3
H7N8	9	H10N4	3
H9N1	9	H10N6	3
H11N1	9	H10N8	3
H2N7	8	H2N1	2
H3N5	8	H2N7	2
H4N3	8	H3N9	2
H1N5	7	H7N6	2
H2N8	7	H10N1	2
H4N9	7	H11N8	2
H6N9	7	H12N4	2
H10N6	7	H13N6	2
H5N9	6	H16N3	2
H6N3	6	H1N5	1
H10N1	6	H1N7	1
H10N8	6	H1N9	1
H13N2	6	H2N5	1
H1N8	5	H4N1	1
H2N5	5	H4N4	1
H3N3	5	H4N5	1
H5N7	5	H4N7	1
H7N4	5	H5N7	1
H9N5	5	H5N9	1
H10N4	5	H6N7	1
H10N5	5	H7N8	1
H11N8	5	H8N5	1
H3N9	4	H8N7	1
H4N7	4	H9N4	1
H5N8	4	H9N5	1
H10N2	4	H9N8	1
H12N4	4	H9N9	1
H1N6	3	H10N2	1
H3N7	3	H10N5	1
H4N5	3	H11N5	1
H6N4	3	H11N6	1
H7N5	3	H12N6	1
H9N6	3	H12N7	1
H11N6	3	H14N5	1
H11N7	3		
H12N3	3		
H12N8	3		
H13N9	3		
H1N7	2		
H2N6	2		
H4N1	2		
H4N4	2		
H9N3	2		
H9N7	2		
H9N9	2		

H10N9	2
H12N1	2
H12N6	2
H13N8	2
H14N8	2
H15N8	2
H15N9	2
H1N4	1
H2N4	1
H5N6	1
H6N7	1
H8N2	1
H8N3	1
H8N8	1
H9N4	1
H9N8	1
H11N5	1
H12N7	1
H12N9	1
H13N3	1
H14N2	1
H14N6	1
H15N4	1

NP

Training dataset		Testing dataset	
H1N1	597	H3N2	137
H3N2	517	H1N1	134
H5N1	432	H5N1	112
H9N2	284	H9N2	78
H6N2	136	H4N6	31
H3N8	110	H5N2	28
H5N2	98	H3N8	27
H4N6	84	H6N2	24
H6N1	77	H6N1	22
Others*	73	Others*	20
H2N2	70	H10N7	18
H7N3	62	H7N3	17
H11N9	57	H2N2	15
H10N7	52	H11N9	14
H7N2	46	H6N6	12
H6N6	42	H7N2	11
H2N3	37	H7N7	11
H7N7	34	H2N3	9
H6N8	31	H5N3	7
H3N6	27	H7N9	7
H7N1	25	H6N8	6
H4N8	24	H8N4	6
H5N3	22	H11N2	6
H11N2	22	H13N6	6
H12N5	22	H2N9	5
H7N9	19	H16N3	5
H8N4	18	H3N6	4
H6N5	16	H4N8	4
H1N2	14	H6N5	4
H2N1	13	H7N1	4
H4N2	12	H1N2	3
H2N9	11	H9N1	3
H13N6	11	H13N9	3
H5N9	10	H3N1	2
H10N3	9	H10N5	2
H11N1	9	H14N6	2
H1N3	8	H1N3	1
H4N3	6	H1N4	1
H7N6	6	H1N7	1
H10N4	6	H2N5	1
H11N6	6	H2N7	1
H11N8	6	H3N7	1
H16N3	6	H4N2	1

H2N7	5	H4N7	1
H3N5	5	H4N9	1
H5N5	5	H5N5	1
H7N8	5	H7N5	1
H10N8	5	H7N6	1
H11N3	5	H9N5	1
H13N2	5	H9N6	1
H3N1	4	H9N9	1
H3N9	4	H10N2	1
H5N7	4	H10N3	1
H9N5	4	H10N4	1
H10N5	4	H11N8	1
H10N9	4	H13N3	1
H1N8	3		
H1N9	3		
H2N5	3		
H3N3	3		
H5N8	3		
H6N4	3		
H7N4	3		
H12N3	3		
H2N8	2		
H3N7	2		
H4N1	2		
H4N4	2		
H4N5	2		
H4N9	2		
H5N6	2		
H6N3	2		
H9N1	2		
H9N9	2		
H10N2	2		
H13N9	2		
H5N4	1		
H6N9	1		
H8N2	1		
H9N6	1		
H9N7	1		
H10N6	1		
H12N4	1		
H12N6	1		
H12N7	1		
H12N8	1		
H13N8	1		
H15N4	1		
H15N8	1		

H15N9	1

NS1

Training dataset		Testing dataset	
H1N1	888	H1N1	229
H5N1	790	H5N1	190
H3N2	774	H3N2	183
H9N2	482	H9N2	111
H5N2	163	H3N8	41
H6N2	151	H5N2	40
H3N8	136	Others*	34
H4N6	123	H4N6	32
H6N1	108	H6N2	30
Others*	106	H6N1	26
H2N2	61	H7N2	18
H7N2	61	H2N2	17
H7N3	59	H10N7	15
H11N9	50	H4N8	13
H6N6	43	H11N9	13
H10N7	43	H6N6	12
H7N7	40	H6N8	12
H6N8	38	H7N3	11
H3N6	32	H7N7	10
H7N1	32	H3N6	9
H2N3	31	H7N1	9
H7N9	30	H5N3	6
H1N2	29	H8N4	6
H4N8	29	H2N3	5
H11N2	23	H4N2	5
H6N5	19	H2N9	4
H8N4	19	H6N5	4
H12N5	19	H10N3	4
H5N3	18	H10N8	4
H13N6	18	H11N3	4
H2N1	11	H1N2	3
H4N2	11	H1N3	3
H16N3	9	H3N7	3
H3N1	8	H4N1	3
H5N9	8	H7N9	3
H2N9	7	H9N1	3
H11N3	7	H11N2	3
H13N9	7	H13N9	3
H4N9	6	H16N3	3
H5N5	6	H2N1	2
H10N3	6	H2N6	2
H11N6	6	H5N5	2
H2N7	5	H5N9	2
H5N8	5	H7N6	2
H7N4	5	H9N5	2
H9N1	5	H11N8	2
H11N1	5	H12N5	2
H1N3	4	H13N6	2
H2N8	4	H1N5	1
H4N3	4	H1N6	1
H5N7	4	H1N9	1
H6N3	4	H2N7	1
H6N4	4	H3N5	1
H7N6	4	H4N5	1
H7N8	4	H4N7	1
H10N6	4	H4N9	1
H11N8	4	H5N8	1
H13N2	4	H7N8	1
H1N9	3	H8N2	1
H3N3	3	H9N8	1
H7N5	3	H9N9	1
H9N5	3	H10N1	1
H10N1	3	H10N4	1
H10N2	3	H10N5	1
H10N5	3	H11N1	1
H1N5	2	H11N4	1
H1N8	2	H11N6	1
H2N5	2	H11N7	1
H3N7	2	H12N3	1
H3N9	2	H12N9	1
H4N4	2	H13N2	1
H6N9	2	H14N2	1
H9N7	2		
H9N8	2		
H9N9	2		
H10N4	2		
H10N9	2		
H11N7	2		
H12N8	2		
H1N4	1		
H2N6	1		
H3N4	1		
H4N1	1		
H4N5	1		
H4N7	1		
H5N6	1		
H6N7	1		
H8N2	1		
H9N4	1		

H9N6	1
H10N8	1
H11N5	1
H12N3	1
H12N6	1
H12N9	1
H13N3	1
H13N8	1
H14N6	1
H15N2	1
H15N8	1

NS2

Training dataset		Testing dataset	
H5N1	319	H1N1	71
H1N1	269	H5N1	62
H9N2	218	H9N2	60
H3N2	187	H3N2	49
H5N2	62	H5N2	18
H6N2	51	H6N1	12
H3N8	46	Others	11
H4N6	45	H3N8	10
Others*	33	H6N6*	8
H6N1	32	H2N2	7
H7N2	32	H4N6	7
H7N3	30	H6N2	5
H11N9	24	H7N1	4
H2N2	23	H7N3	4
H2N3	17	H13N6	4
H7N7	17	H4N3	3
H6N6	16	H6N8	3
H7N9	16	H7N9	3
H10N7	16	H11N9	3
H6N8	15	H1N2	2
H4N8	13	H2N3	2
H1N2	12	H3N6	2
H13N6	11	H5N3	2
H3N6	8	H7N2	2
H7N1	8	H7N7	2
H8N4	8	H10N7	2
H5N3	6	H13N2	2
H16N3	6	H1N5	1
H2N1	4	H2N9	1
H4N2	4	H3N5	1
H6N5	4	H4N8	1
H7N6	4	H4N9	1
H11N2	4	H5N5	1
H11N6	4	H5N8	1
H12N5	4	H5N9	1
H5N5	3	H6N5	1
H5N9	3	H7N6	1
H9N7	3	H8N4	1
H10N9	3	H9N1	1
H13N9	3	H9N8	1
H1N3	2	H11N2	1
H2N9	2	H11N7	1
H5N7	2	H11N8	1
H7N4	2		
H7N5	2		
H7N8	2		
H9N5	2		
H10N3	2		
H11N3	2		
H11N8	2		
H13N2	2		
H1N5	1		
H2N5	1		
H2N6	1		
H2N8	1		
H3N1	1		
H3N5	1		
H3N7	1		
H3N9	1		
H4N3	1		
H4N9	1		
H6N3	1		
H6N4	1		
H6N9	1		
H8N2	1		
H9N9	1		
H10N1	1		
H10N2	1		
H10N4	1		
H10N5	1		
H10N6	1		
H11N7	1		
H12N4	1		
H12N7	1		
H13N3	1		
H13N8	1		
H14N6	1		
H15N4	1		
H12N5	1		
H15N2	1		
H15N8	1		

PA

Training dataset		Testing dataset	
H1N1	1223	H1N1	327
H3N2	922	H3N2	245
H5N1	736	H5N1	198
H9N2	430	H9N2	110
H3N8	255	H4N6	65
H5N2	250	H3N8	62
H4N6	217	Others*	62
Others*	210	H5N2	48
H6N2	186	H6N2	43
H6N1	127	H7N3	32
H10N7	121	H6N1	29
H11N9	109	H11N9	29
H7N2	103	H7N2	25
H7N3	101	H10N7	19
H2N3	84	H2N3	18
H2N2	81	H6N8	18
H6N8	65	H6N6	17
H7N7	64	H7N9	15
H6N6	61	H2N2	13
H3N6	57	H3N6	12
H4N8	55	H1N2	9
H12N5	49	H4N8	9
H8N4	46	H5N3	9
H11N2	45	H8N4	9
H7N9	36	H11N2	8
H7N1	33	H12N5	8
H5N3	32	H2N9	7
H6N5	27	H7N1	7
H4N2	26	H7N7	7
H1N2	21	H13N6	6
H2N1	19	H16N3	6
H2N9	19	H1N3	5
H13N6	16	H4N3	5
H1N9	15	H6N5	5
H16N3	15	H9N1	5
H2N7	14	H2N7	4
H10N3	14	H4N9	4
H1N3	13	H2N1	3
H3N1	13	H3N1	3
H11N1	12	H6N4	3
H10N9	11	H7N6	3
H4N9	10	H10N3	3
H5N9	10	H1N8	2

H7N6	10	H2N5	2
H13N9	10	H3N3	2
H5N5	9	H4N2	2
H6N3	9	H4N5	2
H7N4	9	H5N5	2
H10N6	9	H5N8	2
H11N3	9	H5N9	2
H2N5	8	H6N9	2
H3N5	8	H7N5	2
H10N8	8	H9N5	2
H1N5	7	H9N7	2
H10N1	7	H10N4	2
H11N8	7	H10N5	2
H7N8	6	H11N1	2
H9N1	6	H12N4	2
H10N2	6	H13N2	2
H10N4	6	H13N9	2
H13N2	6	H1N4	1
H5N7	5	H1N5	1
H6N9	5	H1N6	1
H3N3	4	H2N8	1
H3N9	4	H3N5	1
H4N7	4	H3N9	1
H9N9	4	H4N4	1
H10N5	4	H5N4	1
H12N8	4	H6N3	1
H1N6	3	H7N8	1
H1N8	3	H8N2	1
H2N6	3	H9N9	1
H4N5	3	H10N8	1
H5N8	3	H10N9	1
H6N4	3	H11N6	1
H9N5	3	H11N8	1
H11N6	3	H12N6	1
H12N4	3	H14N5	1
H1N4	2	H14N6	1
H1N7	2		
H2N8	2		
H3N7	2		
H4N1	2		
H4N4	2		
H5N4	2		
H5N6	2		
H6N7	2		
H7N5	2		
H9N6	2		

H11N7	2
H12N1	2
H12N3	2
H12N6	2
H12N9	2
H13N3	2
H14N2	2
H14N8	2
H2N4	1
H4N3	1
H8N8	1
H9N4	1
H9N7	1
H9N8	1
H13N8	1
H14N5	1
H15N4	1
H15N9	1

PB1

Training dataset		Testing dataset	
H1N1	1124	H1N1	295
H3N2	856	H3N2	203
H5N1	622	H5N1	162
H9N2	335	H9N2	84
H3N8	201	H5N2	61
H5N2	197	H3N8	50
H6N2	188	H6N2	40
H4N6	176	Others*	38
H6N1	125	H4N6	37
Others*	98	H6N1	23
H7N3	96	H11N9	23
H10N7	90	H10N7	20
H11N9	76	H2N3	19
H2N2	66	H7N3	18
H7N2	61	H6N6	17
H6N8	58	H7N2	17
H2N3	50	H7N7	16
H4N8	45	H2N2	13
H6N6	45	H6N8	12
H7N7	42	H8N4	11
H11N2	36	H1N2	10
H3N6	35	H3N6	10
H12N5	32	H4N8	10
H5N3	29	H4N2	6
H7N1	28	H7N1	6
H6N5	26	H2N1	5
H7N9	24	H7N6	5
H8N4	24	H13N6	5
H4N2	21	H2N9	4
H1N2	19	H5N3	4
H2N9	17	H5N7	4
H2N1	14	H10N8	4
H13N6	14	H11N2	4
H16N3	14	H1N3	3
H1N3	11	H2N7	3
H10N3	11	H3N3	3
H11N1	11	H7N9	3
H2N7	10	H12N5	3
H11N3	10	H16N3	3
H5N5	9	H1N8	2
H6N4	9	H3N9	2
H7N6	9	H4N4	2
H2N5	8	H5N9	2

H3N1	8	H6N5	2
H9N1	8	H6N9	2
H10N4	8	H7N4	2
H5N9	7	H10N2	2
H9N9	7	H11N3	2
H10N1	7	H11N8	2
H10N2	7	H1N6	1
H1N9	6	H3N4	1
H3N5	6	H3N5	1
H4N9	6	H4N1	1
H5N8	6	H4N3	1
H9N5	6	H4N7	1
H10N9	6	H5N5	1
H13N9	6	H5N6	1
H6N3	5	H7N8	1
H7N4	5	H8N2	1
H7N8	5	H9N1	1
H10N6	5	H9N5	1
H11N6	5	H9N8	1
H13N2	5	H10N1	1
H1N5	4	H10N3	1
H4N3	4	H10N4	1
H4N5	4	H10N5	1
H10N8	4	H10N6	1
H11N8	4	H10N9	1
H1N6	3	H11N6	1
H2N8	3	H12N4	1
H3N7	3	H12N7	1
H3N9	3	H12N8	1
H4N7	3	H13N2	1
H9N7	3	H13N3	1
H12N4	3	H13N8	1
H12N6	3	H13N9	1
H1N7	2		
H2N6	2		
H3N3	2		
H4N1	2		
H4N4	2		
H5N4	2		
H5N6	2		
H6N9	2		
H7N5	2		
H9N4	2		
H9N6	2		
H10N5	2		
H11N7	2		

H12N3	2
H12N9	2
H14N6	2
H1N4	1
H1N8	1
H2N4	1
H5N7	1
H8N8	1
H9N8	1
H11N5	1
H12N8	1
H13N3	1
H14N5	1
H14N8	1
H15N4	1

PB1-F2

Training dataset		Testing dataset	
H3N2	531	H3N2	114
H5N1	253	H1N1	69
H9N2	248	H5N1	69
H1N1	219	H9N2	62
H3N8	180	H4N6	44
H4N6	142	H3N8	41
Others*	120	H5N2	27
H5N2	95	Others*	26
H6N2	78	H2N3	20
H11N9	75	H10N7	20
H7N2	72	H6N2	19
H7N3	64	H7N3	18
H10N7	64	H6N1	17
H2N3	54	H11N9	16
H6N1	52	H7N2	15
H2N2	45	H6N6	14
H6N8	43	H3N6	13
H7N7	41	H6N8	10
H11N2	35	H12N5	10
H4N8	34	H4N8	9
H3N6	27	H7N7	9
H6N6	25	H2N2	6
H12N5	25	H1N2	5
H8N4	23	H2N1	5
H4N2	22	H2N9	5
H7N9	21	H7N1	5
H5N3	18	H8N4	5
H1N2	16	H4N2	4
H6N5	13	H6N5	4
H7N1	12	H10N3	4
H1N3	10	H11N2	4
H2N7	10	H13N6	4
H2N9	10	H1N9	3
H2N1	9	H5N8	3
H10N3	9	H7N6	3
H11N1	9	H16N3	3
H13N6	9	H1N3	2
H16N3	9	H1N8	2
H1N9	8	H2N5	2
H3N1	8	H2N7	2
H5N9	8	H3N1	2
H10N9	8	H3N5	2
H3N3	7	H4N7	2
H4N3	7	H5N3	2
H5N5	7	H7N9	2
H11N3	7	H10N1	2
H3N5	6	H10N2	2
H6N3	6	H10N8	2
H10N2	6	H11N1	2
H4N4	5	H13N2	2
H7N4	5	H13N9	2
H9N1	5	H1N5	1
H10N4	5	H1N6	1
H1N5	4	H2N8	1
H1N8	4	H4N5	1
H2N5	4	H4N9	1
H4N5	4	H5N5	1
H4N9	4	H5N9	1
H7N8	4	H6N9	1
H9N5	4	H7N4	1
H10N1	4	H8N2	1
H10N6	4	H8N8	1
H13N2	4	H9N6	1
H13N9	4	H9N7	1
H3N7	3	H9N9	1
H3N9	3	H10N6	1
H6N4	3	H11N3	1
H6N9	3	H11N8	1
H7N5	3	H12N6	1
H7N6	3	H12N7	1
H11N8	3	H12N9	1
H2N6	2		
H3N4	2		
H4N1	2		
H5N4	2		
H5N6	2		
H5N7	2		
H5N8	2		
H9N8	2		
H10N5	2		
H11N6	2		
H12N4	2		
H12N6	2		
H14N8	2		
H1N4	1		
H1N6	1		
H2N4	1		
H2N8	1		
H4N7	1		

H6N7	1
H9N4	1
H9N6	1
H10N8	1
H11N5	1
H11N7	1
H12N9	1
H13N3	1
H14N5	1
H14N6	1
H15N4	1
H15N9	1

PB2

Training dataset		Testing dataset	
H1N1	1293	H1N1	330
H3N2	835	H3N2	221
H5N1	634	H5N1	184
H9N2	324	H9N2	89
H3N8	214	H5N2	62
H5N2	212	H3N8	54
H6N2	187	Others*	45
Others*	167	H4N6	44
H4N6	160	H6N2	42
H6N1	121	H6N1	28
H7N3	96	H7N3	28
H10N7	94	H6N6	21
H11N9	85	H2N2	20
H2N2	68	H7N2	19
H7N2	65	H10N7	19
H6N8	59	H4N8	16
H2N3	58	H11N9	14
H6N6	54	H6N8	13
H7N7	48	H12N5	12
H3N6	38	H7N7	11
H8N4	37	H4N2	10
H5N3	35	H7N9	10
H4N8	34	H11N2	10
H7N9	34	H2N3	9
H7N1	33	H2N9	9
H11N2	33	H7N1	9
H12N5	28	H8N4	7
H1N2	23	H3N6	6
H4N2	23	H11N1	6
H6N5	22	H2N1	5
H2N9	21	H2N7	4
H2N1	16	H5N3	4
H10N3	16	H1N9	3
H3N1	15	H5N9	3
H1N3	13	H6N3	3
H1N9	13	H6N5	3
H7N6	13	H7N6	3
H16N3	11	H11N3	3
H13N6	10	H13N6	3
H11N3	9	H16N3	3
H3N5	8	H1N2	2
H5N5	8	H1N3	2
H5N9	8	H3N1	2
H11N1	8	H7N8	2
H2N7	7	H9N6	2
H4N9	7	H10N4	2
H10N1	7	H10N6	2
H2N5	6	H10N8	2
H4N3	6	H11N8	2
H7N8	6	H1N6	1
H9N1	6	H2N5	1
H10N4	6	H3N3	1
H11N8	6	H3N5	1
H1N5	5	H4N1	1
H3N3	5	H4N4	1
H4N1	5	H4N7	1
H6N3	5	H4N9	1
H10N6	5	H5N5	1
H10N8	5	H5N6	1
H13N2	5	H5N7	1
H13N9	5	H6N9	1
H2N8	4	H7N4	1
H4N5	4	H9N1	1
H9N5	4	H9N7	1
H10N5	4	H9N9	1
H11N6	4	H10N2	1
H12N4	4	H10N5	1
H1N6	3	H10N9	1
H1N8	3	H11N6	1
H3N7	3	H12N7	1
H3N9	3	H13N9	1
H6N4	3		
H6N9	3		
H7N4	3		
H9N8	3		
H9N9	3		
H10N2	3		
H10N9	3		
H12N6	3		
H12N8	3		
H1N7	2		
H2N6	2		
H3N4	2		
H4N4	2		
H5N7	2		
H6N7	2		
H7N5	2		
H11N5	2		
H11N7	2		

H12N3	2
H12N9	2
H14N6	2
H14N8	2
H1N4	1
H2N4	1
H4N7	1
H5N4	1
H5N6	1
H5N8	1
H8N8	1
H9N4	1
H9N7	1
H13N8	1
H14N5	1
H15N2	1
H15N4	1
H15N8	1
H15N9	1
